# On Dysmenorrhœa

**Published:** 1872-06

**Authors:** Lombe Atthill

**Affiliations:** Dublin, Fellow and Examiner in Midwifery King and Queen’s College of Physicians, Obstetric Physician to the Adelaide Hospital, Dublin


### ON DYSMENORRHŒA.

BY LOMBE ATTHILL, M. D., DUBLIN,

Fellow and Examiner in Midwifery King and Queen's College of Physicians, Obstetric Physician to the Adelaide Hospital, Dublin.

For practical purposes Dr. Atthill thinks it sufficient to class cases of dysmenorrhœa under three heads—namely: 1st, spasmodic; 2d, inflammatory; and 3d, mechanical dysmenorrhœa.

In spasmodic dysmenorrhœa the pain, as in other forms, precedes the appearance of the discharge. In the majority of cases it is met with either in delicate girls of feeble constitution and leuco-phlegmatic temperament, or again in women of full habit, especially if they lead an inactive life. The flow is in general scanty and its appearance does not bring any marked relief, the pain continuing more or less during the whole of the period; it is not, however, always equally severe, but is paroxysmal, being less so while the patient is warm, but becoming aggravated by the least exposure to cold. This form of dysmenorrhœa is by some writers described as neuralgic; its true nature, however, is very obscure, but its attacks can almost with certainty be cut short by the administration of sedatives and antispasmodics. Dr. Atthill generally gives a pill containing half a grain of opium, one of Indian hemp, and two of camphor, at bedtime—a combination which seldom fails to give at least temporary relief, or if for any reason opium is objectionable, he substitutes for it two grains of the extract of conium.

When the attacks have become habitual, and the patient is consequently obliged to have recourse regularly to the use of medicines to obtain relief, Dr. Atthill usually directs her to have by her for ready use, a mixture containing two drachms of sulphuric ether, half a drachm of liquor opii sedatives, three drachms of the tincture hyoscyamus, one drachm of the hydrate of chloral, two drachms of the spirits of chloroform, and water sufficient to make a six-ounce mixture. Of this she should take a tablespoonful every two hours. She should bathe the feet at bed-time, and if prevented by the pain from sleeping, take a full dose of the hydrate of chloral. This treatment is, however, only palliative, and as the cause generally lies in some fault of the constitution or system at large, our object should be to correct that condition by treatment carried out during the interval between the menstrual periods. Dr. Atthill is convinced that many cases of spasmodic dysmenorrhœa are due to congestion of the lining membrane of the uterus, and that this is specially the case in women of full habit who lead indolent lives, and in whom great benefit follows the adoption of more abstemious diet and more active habits, together with occasional use of saline purgatives.

Inflammatory or congestive dysmenorrhœa is a common affection, and the sufferings due to it are often very acute; the pain, however, is generally relieved by the appearance of the menstrual flow—a fact capable of easy explanation, for the loss of blood relieves the congestion which has existed, just as it would a similar condition existing in any other part of the body. In this form the uterus, or at least its lining membrane, is in a state of chronic inflammation, sometimes also there is associated with it an ulcerated condition of the cervical canal; sexual intercourse is generally painful, this being due to extreme sensibility of the cervix, a not uncommon result of chronic inflammation of that part of the womb. In the spasmodic form of dysmonorrhœa the pain is nearly always referred to the back, or to the lower portion of the abdomen. In inflammatory dysmenorrhœa, on the other hand, it is often more intense along the edge of the false ribs on the left side, shooting up to the shoulder, and down to the ovary of that side; pressure too over the ovary causes pain.

The treatment of inflammatory dysmenorrhœa includes three indications:

1st. The removal of all causes keeping up the existing irritation. Foremost among these is the abstinence from sexual intercourse, for not only does the act itself generally cause pain and therefore must be injurious, but the occurrence of conception is to be specially avoided. Horse exercise, fatiguing walks, or even household occupations which necessitate much standing, should be given up, while the occurrence of constipation is to be guarded against.

2d. The inflammatory condition of the uterus is to be relieved by local depletion either by means of leeches applied before the menstrual period or by puncturing the cervix uteri and encouraging the bleeding. It is not suitable to the case of young unmarried girls, as it necessitates the use of the speculum; in them the leeches should be applied to the inside of the thighs, but in married women to the cervix uteri itself. Mild purgatives should also be from time to time administered. When by these means we have succeeded in relieving the congestion of the uterus, considerable benefit will be derived from blisters applied over the sacrum or to the abdomen a little above the pubes.

3d. If the case be of long standing, and the symptoms, though relieved, do not entirely disappear, showing that a certain amount of endometritis still exists, Dr. Atthill recommends us to cauterize the cervical canal, and even in many cases the whole interior of the uterus, with strong nitric acid. Another method of relieving these forms of painful menstruation depending on chronic inflammation of the uterus, is the use of glycerine. It is not suitable to the early stages of the affection, but it often answers admirably after more active treatment; it is specially useful if, from the presence of ulceration or any other cause, any strong caustic has been applied to the cervix, and as sometimes happens, an unhealthy irritatable sore remains. In such cases a pledget of cotton soaked in glycerine and introduced into the vagina will, in twenty-four hours, perfectly clean the sore, while the copious watery discharge which it produces will greatly relieve the local congestion. In chronic cases the injection of a few drops of the pure glycerine into the cavity of the uterus two or three times a week, as recommended by Dr. Marion Sims, is very useful. Dr. Atthill has met with but little benefit from the exhibition of medicines in inflammatory dysmenorrhœa. Where ovarian excitement exists, bromide of potassium in twenty-grain doses three times a day, sometimes does good; the bichloride of mercury, in small doses and continued for a considerable time, has been recommened by several writers, but it has disappointed the author's expectations. Purgatives, especially the saline, seem the only medicines capable of producing real benefit; these, to do good, should be exhibited just before the menstrual period.

Dr. Atthill next considered those forms of dysmenorrhœa which depend on mechanical causes. Of these there are three varieties—namely: those in which the cervical canal is so flexed as to obstruct the escape of the menstrual discharge; secondly, those in which inflammation or congestion of the lining membrane exists to such extent as to cause temporary closure of the canal or of the os internum; and thirdly, those in which, from some congenital malformation, or acquired cause, the os internum or the cervical canal throughout its entire length is permanently narrow and constricted. To this last may be added those cases in which fibrous tumors are met with in connection with and often probably causing dysmenorrhœa.

Painful or difficult menstruation is frequently met with in women in whom the uterus is flexed; but though flexions of the uterus may and certainly do sometimes interfere with the exit of the menstrual flow, they certainly seldom do so unless the flexion be complicated by the existence of chronic inflammation or the presence of a fibroid. In such cases we should certainly endeavor to relieve the flexion, and see if by placing the fundus in its normal position, and supporting it there by a pessary, we can relieve the patient before having recourse to surgical means, which are less suitable in this than in any of the other forms of mechanical dysmenorrhœa. In cases of inflammatory swelling of the lining membrane of the uterus, in which the os internum or some portion of the cervical canal becomes so narrowed in consequence of the tumefaction of the parts as to present a mechanical impediment to the discharge of the menses, in such cases, if the treatment already recommended fail, Dr. Atthill has no hesitation in having recourse to surgical treatment with the view of procuring relief; indeed, it is obvious that an operation which divides the cervix so freely as does that introduced by Sir James Simpson, must be calculated to give permanent relief to the congested organ, but the operation should not be had recourse to till other means have failed, including the dilatation of the cervix by means of sea-tangle tents. Dr. Atthill unhesitatingly condemns the use of any of the metal instruments which have been suggested for the purpose of dilating the cervix; their use is attended with danger, as they act too rapidly and sometimes rupture the uterine fibres; several cases of severe inflammation and even of death are on record as following their use, while the sea-tangle is perfectly harmless.—*Medical Press and Circular.*

